# Traditional vegetable preservation technologies practiced in Acholi subregion of Uganda improves mineral bioavailability but impacts negatively on the contribution of vegetables to household needs for micronutrients

**DOI:** 10.1002/fsn3.1931

**Published:** 2021-01-07

**Authors:** Raymond Bighaghire, Lawrence Okidi, Christopher Muggaga, Duncan Ongeng

**Affiliations:** ^1^ Department of Food Science and Postharvest Technology Faculty of Agriculture and Environment Gulu University Gulu Uganda

**Keywords:** household micronutrient requirements, nutrient bioavailability, nutrient losses, traditional African vegetable preservation

## Abstract

The impact of traditional African preservation methods on the contribution of vegetables to household micronutrient needs (Recommended Dietary Allowance: RDA) has largely remained unquantified. Using Acholi subregion of Uganda as a case area, this study examined using the predominant vegetables consumed in fresh and preserved forms (cowpeas—*Vigna unguicullata*, okra/lady fingers—*Abelmoschus esculentus*, Malakwang—*Hibscus cannabinus*, and eggplants—*Solanum melongena*), the effect of major traditional vegetable preservation methods (sun drying, boiling and sun drying, and salting and sun drying) on the contents of micronutrients (vitamin A, iron, zinc, calcium, magnesium, and phosphorus), the levels of antinutritional factors (total polyphenols, oxalate, tannins, and phytate), bioavailability of iron and zinc, and the contribution of vegetables to the cumulative annual household RDA for micronutrients. Laboratory analysis showed that all the preservation methods, except the sun drying method reduced the contents of micronutrients by 20%–82% (*p* ≤ .05). The contents of antinutritional factors reduced by 1%–80% while in vitro bioavailability of iron and zinc increased by 21%–296% (*p* ≤ .05). Nutritional computation revealed that except for calcium, the preservation methods combined, reduced the contribution of the vegetables to cumulative annual RDA for other micronutrients by 28%–60%. These results demonstrate that improvements in bioavailability of essential nutrients (iron and zinc) by traditional preservation methods investigated are associated with significant loss of micronutrients which culminates in significant reduction in the contribution of cultivated vegetables to household RDA for micronutrients. Traditional African preservation methods should be optimized for nutrient retention.

## Graphical abstract

This study examined the effect of traditional African vegetable preservation methods on the contribution of vegetables to household needs for micronutrients. The results showed that the traditional methods improve mineral bioavailability but negatively affects the contribution of vegetables to household mironutrient needs due to excessive loss of nutrients.

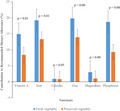

## 1 INTRODUCTION

The significance of adequate consumption of fruits and vegetables to human health and nutrition has received great attention globally. This is largely because of their high contents of micronutrients and bioactive compounds that are essential for nutrition and health (Arimond et al., 2018; Sivakumar et al., 2018). The increase in occurrence and prevalence of diet‐associated noncommunicable diseases such as cancer, diabetes, obesity, and high blood pressure has also created renewed attention to the need for adequate consumption of fruits and vegetables (Lin et al., 2018; Rinaldo, 2020). As a strategy to meet micronutrient requirements and to contribute to achieving other health outcomes (e.g., reduced level of cholesterol, blood pressure maintenance, weight control), the Food and Agriculture Organization (FAO) and World Health Organization (WHO) recommends that adults should consume at least 400 g of fruits and vegetables per day (FAO & WHO, 2004). Despite the FAO and WHO recommendation on fruits and vegetables intake, micronutrient deficiency is still an important nutritional constraint, most especially in developing regions of the world and rural areas in particular (Fongar et al., 2019). sub‐Saharan Africa ranks highest in terms of micronutrient deficiency. This is well evidenced by statistics which show deficiency rates of 29.1% for vitamin A (Bailey et al., 2015), 43% for iron (Engle‐stone et al., 2017) and 26% for zinc (Wessells & Brown, 2012) in the subcontinent.

In many sub‐Saharan African countries such as Uganda, because of poverty and less functional market systems, rural households rely largely on own production to support food needs (Akombi et al., 2017). In most situations, such households depend on vegetables because grain stocks hardly support year‐round food needs while staple food available in the market is often too expensive for them to afford (Boukary et al., 2016). On the other hand, and also due to poor socio‐economic conditions, resource‐constrained households in the subcontinent, majority of which are concentrated in rural areas, hardly access animal source foods such as meat and meat products, and poultry and poultry products (Boukary et al., 2016). This is notwithstanding the fact that animal‐based foods are a good source of essential micronutrients with high degree of bioavailability (Akomo et al., 2016). These situations indicate the importance of vegetables, which are easily cultivated and take a short time to mature (Rybak et al., 2018), to household nutrition and micronutrient intake in particular, among poor rural communities in the subcontinent.

Vegetables contain substantial quantities of micronutrients such as vitamin A, iron, calcium, zinc, magnesium, and phosphorus, and when consumed in adequate amounts as recommended by WHO (2003), can contribute effectively to prevention and alleviation of micronutrient‐associated medical conditions such as night blindness, iron deficiency disorders, osteoporosis (weak bones), and weak teeth (Akomo et al., 2016). Other than micronutrients, vegetables are also a good source of fiber and a variety of non‐nutrient phytochemicals (such as phenols, phytates, saponins, oxalates, and tannins), when consumed in small quantities, are believed to confer health benefits including anti‐inflammatory, antioxidant, anticancer, lipid lowering, and blood pressure regulation actions (Lin et al., 2018). However, these phytochemicals, when present in large quantities, confer antinutritional properties and thus become antinutritional or antinutrient factors instead. These antinutrient factors, through their direct action and or via their metabolic products reduce bioavailability of essential nutrients such as iron, calcium, zinc, and protein (Bazaz et al., 2016; Burgos et al., 2018; Gwamba et al., 2019).

In many sub‐Saharan African countries such as Uganda, production of vegetables is seasonal due to seasonal agricultural patterns dictated by weather. Therefore, fresh vegetables are largely unavailable during off‐season periods. Seasonal nature of agricultural production and high level of perishability hinder year‐round availability of fresh vegetables. In addition, the presence of antinutritional factors in them also reduces micronutrient bioavailability (Samtiya et al., 2020). While preservation generally solves the problem of perishability and availability (Shen et al., 2018), it inevitably affects nutritional and antinutritional contents to various extents (Gwamba et al., 2019) and the extent largely depends on the preservation method used (Bulbula & Urga, 2018). Information on the impact of food preservation on nutritional quality of vegetables is largely based on experiments conducted under controlled laboratory conditions (Adeleke et al., 2017; Burgos et al., 2018; Gwamba et al., 2019).

In the context of rural development and in the context of developing countries such as those in sub‐Saharan Africa, results derived from such laboratory‐controlled studies may not be easily applied because of technological lacuna in rural areas. In practice, rural communities in such countries largely apply traditional methods to preserve vegetables (Rybak et al., 2018; Santhi & Sengottuvel, 2016). However, limited information exists on how African traditional vegetable preservation methods as practiced under rural conditions affect the contribution of vegetables to household requirements (RDA) for micronutrients. This question partly has implications on outcomes of micronutrient nutrition among African rural communities. The likelihood of the impact draws credence from undesirable statistics on micronutrient intake in African countries. For instance, among rural households in Uganda, deficiency rates of essential micronutrients are high and stand at 15.8% for vitamin A, 54% for iron, and 31.7% for calcium among children (Nabeta et al., 2015; UBOS & ICF, 2018). Therefore, using Acholi subregion of Uganda as a case area, the objective of this study was to examine under real rural set‐up, based on the predominant vegetables that are consumed in fresh and preserved forms, the effect of traditional vegetable preservation methods on the contents of micronutrients, the levels of antinutritional factors, mineral bioavailability, and the contribution of vegetables to the cumulative annual household RDA for micronutrients.

## 2 MATERIALS AND METHODS

### 2.1 Study area

**FIGURE 1:**
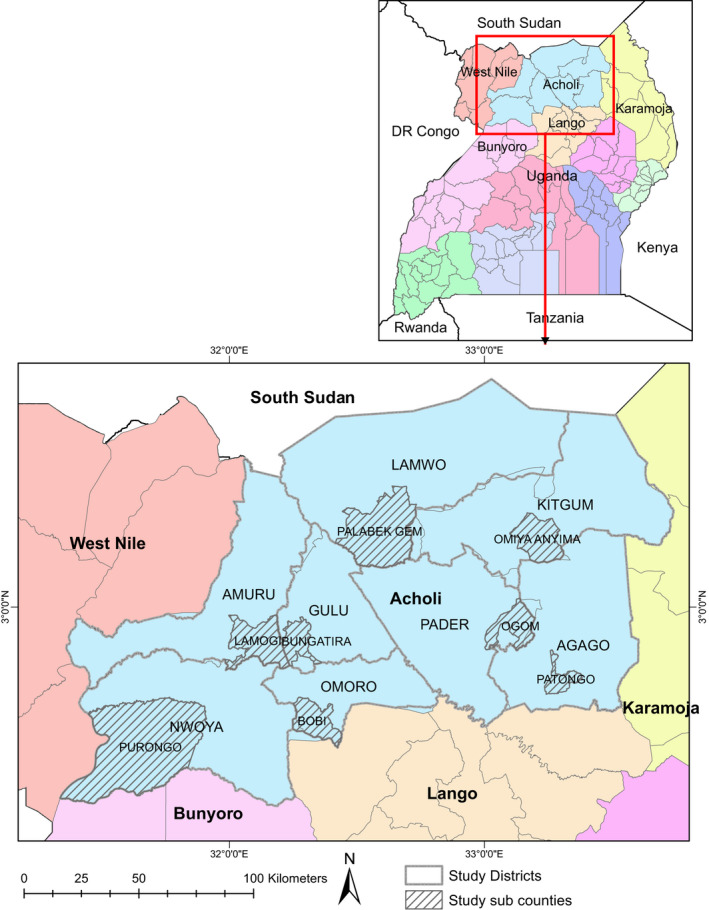
Map indicating subcounties, parishes, and villages where the study was conducted

The study was conducted in Acholi subregion of Uganda. The subregion was purposively selected because of the high state of micronutrient deficiency rates experienced in the recent past (UBOS & ICF, 2018), and previous studies on vegetable consumption had been carried out in it (Anywar et al., 2014; Okidi et al., 2018). The subregion is made up of eight districts (Gulu, Kitgum, Agago, Pader, Amuru, Nwoya, Omoro, and Lamwo). The main agro‐ecological zone in the subregion is savannah grassland characterized by wet and dry seasons with an annual rainfall of approximately 1,500 mm (ACF, 2007). The main economic activity in the subregion is agriculture with over 90% of the population engaged in subsistence farming (UBOS, 2016). The main crops cultivated are maize, sesame, cassava, beans, rice, and a variety of vegetables. The subregion has an area of 28,500 km^2^ and a population size of about 1,500,770 people (UBOS, 2016). The subregion is bordered by South Sudan to the north, Karamoja subregion to the east, Bunyoro subregion to the west, West Nile subregion to the southwest, and Lango subregion to the south (Figure 1).

### 2.2 Identification and selection of vegetables and preservation methods

Focus group discussions (FGD) were conducted to catalog and select traditional technologies largely used in preservation of vegetables and the vegetables that are normally preserved in the community. Eight FGDs, one session in one village for each of the eight districts that comprise Acholi subregion (Lawiye adul village, Gulu district; Itoduny East village, Pader district; Owinykibul village, Agago district; Pawatomero village, Nwoya district; Abam village, Lamwo district; Lwalakwar village, Amuru district; Onekdyel village, Omoro district; and Amoyocol village, Kitgum District, see Figure 1) were conducted. This number of FGD is consistent with the results of Guest et al. (2006) which showed that 6–12 FGD sessions are sufficient for saturation of information in qualitative studies. Each FGD session had 8–12 members and lasted 1.5–2 hr. Participants in the FGD exercise were selected purposively based on their knowledge in cultivating and preserving vegetables. The FGDs were managed by facilitators with knowledge of local language (Luo/Acholi) and culture of the area. Before conducting the FGDs, facilitators were trained on how to administer them. However, in constituting the FGD members, homogeneity (individuals with similar livelihoods within each group) was taken into account to capitalize on the participants' shared experiences and make individual participants not to be intimidated by other group members (Wong, 2008).

The FGD exercise revealed that sun drying, boiling and sun drying, and salting and sun drying, smoking, air drying, and fermentation are the traditional preservation methods available in the community. From this catalog of preservation methods, the community identified and agreed that sun drying, boiling and sun drying, and salting and sun drying are the most common methods usually applied for preserving vegetables in the community. The FGDs further identified cowpeas (*Vigna unguicullata*), okra/lady fingers (*Abelmoschus esculentus*), Malakwang (*Hibscus cannabinus*), eggplants (*Solanum melongena*), cabbages (*Brassica oleracea*), beans (*Phaseolus vulgaris*), green bell and red peppers (*Capsicum* spp), and dodo (*Amaranths*) as the vegetables that are usually preserved. In‐depth investigation during FGD sessions revealed that, of all the vegetables that can potentially be preserved, *V. unguicullata*, *A. esculentus*, *H. cannabinus*, and *S. melongena* were the vegetables usually preserved using traditional preservation technologies prioritized above. On the basis of the forestated FGD process, the vegetables, *V. unguicullata*, *A. esculentus*, *H. cannabinus*, *S. melongena*, and the traditional preservation methods, sun drying, boiling and sun drying, and salting and sun drying, were chosen for use in the study.

### 2.3 Processing of selected vegetables according to selected preservation methods

Processing of *V. unguicullata*, *A. esculentus*, *H. cannabinus*, and *S. melongena* using sun drying, boiling and sun drying, and salting and sun drying was conducted in the community making use of the expertise of women who had at least 5 years of experience. For each vegetable, processing was conducted in each of the villages where FGD took place, giving a replicate size of eight. For each location, freshly harvested samples of each vegetable were bought from four households and mixed together to get a representative sample according to Mbah et al. (2012). Each fresh vegetable sample from each village was divided into four portions. Portions of each of the fresh samples, used as a control, were separately put in high‐density polythene bags (HDPE) and immediately taken to the laboratory where they were washed, cut into small pieces with knife, and divided into two subdivisions. Samples in one set of subdivision were dried using hot air oven (AAF‐6701; Shimadzu Corp.) at 105°C for 3 hr (AOAC, 1990) in order to determine the dry matter content. Samples in the second subdivision were used for determination of contents of micronutrients, antinutritional factors and in vitro nutrient bioavailability as described under Section 2.4 and 2.5. The other three portions of each vegetable were subjected to the three preservation methods (sun drying, boiling and sun drying, and salting and sun drying) selected following the FGD process described under Section 2.2.

Sun drying involved cutting the vegetable's samples into small pieces (thickness of about 1–2 cm), spreading them uniformly on transparent plastic material, and drying under the sun for 8 hr per day for 3 days. Boiling and sun drying method involved heating vegetable samples for 5–15 min at a temperature range of 95–100°C, followed by cutting them into pieces and then dried as described for the sun drying method. Salting and sun drying method involved immersion of uncut vegetables in 20% (table) salt solution for 15–30 min at ambient condition followed by cutting and sun drying as already described. The treated samples were also packed individually in HDPE bags and taken to the laboratory for further processing. In the laboratory, treated samples were crushed into powder using a mortar with a pestle (Minifuge, Heraeus‐Christ GMBH) and passed through a No. 30 mesh sieve (Minifuge, Heraeus‐Christ GMBH) and thoroughly mixed well into homogenous mixture. The samples were stored in tightly stoppered bottles for further analyses as described in the subsequent subsections.

### 2.4 Analysis of micronutrients

The following micronutrients were analyzed: vitamin A, iron, zinc, calcium, magnesium, and phosphorus. Vitamin A, iron, zinc, and calcium were selected because of their public health importance (Stevens et al., 2013; WHO, 2020). Although phosphorous and magnesium are largely not considered among micronutrients of public health importance, they were included in the study due to the fact that high concentration of phosphorous in the diet impairs calcium metabolism whereas high concentration of magnesium impacts negatively on iron absorption (Lamberg‐Allardt & Kemi, 2017; Sugimoto & Yamada, 2019), thus indirectly making them significant in micronutrient nutrition in developing countries. Vitamin A was analyzed through detection of beta carotene using high‐performance liquid chromatography according to Fatimah et al. (2014) and expressed as retinol equivalent (RE). Iron, calcium, and zinc were each determined using the atomic absorption spectrophotometric (AAS) method making use of the standard curve procedure as previously applied by Norhaizan and Nor Faizadatul Ain (2009). The AAS 500 (PG Instruments Limited) was used upon calibration using standard stock solutions of iron, calcium, and zinc made from AAS Grade Chemicals (Sigma, USA). Absorbance was measured at 248.3, 422.7, and 213.9 nm for iron, calcium, and zinc, respectively (Irungu et al., 2018). Determination of magnesium was performed using the Ethylenediamine‐tetraacetic acid (EDTA) procedure previously reported by Shokrollahi et al. (2016). Phosphorous was determined spectrophotometrically at 880 nm according to the AOAC (2005) method 970.39. The UV‐visible spectrophotometer from CECIL Instruments was used. The mineral elements were each expressed as mg/100 g of the sample.

### 2.5 Analysis of antinutritional factors and mineral bioavailability

The following antinutritional factors were analyzed: total polyphenols, tannins, total oxalates, and phytates. These antinutritional factors were selected because they are the major types that affect bioavailability of nutrients from plant‐based foods (Gwamba et al., 2019; Nadeem et al., 2010; Sinha & Khare, 2017). The content of total phenols was determined spectrophotometrically at 725 nm according to the method previously applied by Burgos et al. (2018) and Škerget et al. (2005), and the results expressed as μg of gallic acid equivalents per gram of dry mass (DM) of the extract (μg GAE/gDM). The content of tannins was also determined spectrophotometrically but using the Folin–Denis quantitative method as previously applied by Ejikeme et al. (2014). Tannic acid (Sigma‐Aldrich Chemicals) was used to prepare the calibration curve while optical density was measured at 700 nm using the Spectrum 23A spectrophotometer (AAF‐6701; Shimadzu Corp.). The concentration of tannins was expressed as tannic acid (mg/100 g). Total oxalate content was determined using ion exchange HPLC chromatographic method previously applied by Savage and Dubois (2006). The standard curve (range 1–20 mg/100 ml) was developed using oxalic acid (Sigma‐Aldrich). Chromatographic separation was carried out using a 300 × 7.8 mm Rezex ROA ion exclusion organic acid column (Phenomenex) attached to a cation H^+^ guard column (Bio‐Rad) held at 25°C. The mobile phase used was an aqueous solution of 25 mM Sulphuric acid. Phytate content was determined using the colorimetric method (Latta & Eskin, 1980). Finally, bioavailability of iron and zinc was determined using in vitro digestion method as previously applied by Kumar et al. (2017) and Kiers et al. (2000).

### 2.6 Contribution of fresh and preserved vegetables to household micronutrient requirements

A cross‐sectional survey employing individual household questionnaire was applied. It involved participants' recall of the quantity of vegetables that were consumed in fresh or in preserved form over a consumption period stretching from April 2017 to March 2018. Participants were women and were purposively selected because they are usually involved in food preparation at home in rural settings (Doss et al., 2018; Johnston et al., 2018). The sample size (*n*) of the households that participated in the study was determined using sample size table for a given population according to Krejcie and Morgan (1970) considering household population of 296,335 households in Acholi subregion (*N* = 296,335). Based on the sample size table, a sample size of 382 households was considered representative of Acholi subregion. To select the participating households, a multi‐stage sampling procedure was used. First, one subcounty was randomly selected from each of the eight districts followed by random selection of two parishes from each of the selected subcounty and one village from each of the selected parish. Finally, 23–24 households were randomly selected from each village. Table 1 provides information on the subcounties, parishes, and villages from which participating households were selected.

**TABLE 1:** A matrix showing subcounties, parishes, and villages where the study was conducted

District	Subcounty	Parish	Villages
Amuru	Lamogi	Gira gira	Opok
		Lacor	Lwalakwar
Gulu	Bugantira	Laliya	Lawiye adul
		Pabwo	Kulu keno
Nwoya	Purongo	Paromo	Belkech
		Pawatomero	Pawatomero west
Omoro	Bobi	Paidwe	Onekdyel
		Palenga	Iraa
Kitgum	Omiya anyima	Melong	Kumele‐wicere
		Tella	Amoyocol
Pader	Ogom	Ogom	Itoduny
		Otong	Wiraa East
Agago	Patongo	Lukwangole	Owinykibul
		Lakwa	Akomo
Lamwo	Palabek gem	Moroto	Kamama central
		Cobo	Abam

Individual household questionnaire was used to collect data on consumption of vegetables identified under Section 2.2. The questionnaire was structured to collect data on amounts produced, amounts consumed, frequency with which they were consumed, duration over which they were consumed, and overall consumption in a year, clearly segregated by duration for which fresh or preserved forms were consumed. In addition, provision was provided for information on household composition by number, age, physiological state, and gender. To ensure data quality, the questionnaire was pretested among 82 households in Alede and Obiya villages in Gulu district, and adjusted for clarity. The Alede and Obiya villages were not selected for the actual study to avoid bias. Actual data collection took place in March 2018 using four research assistants that were trained on individual household method (IHM) for food security (Seaman & Petty, 2009). During data collection, a food weighing scale was used to provide accurate estimate of the quantities of vegetables consumed in each household. All the data collected was checked and verified daily for completeness and eligibility.

### 2.7 Data analysis

One‐way ANOVA was used to determine the effect of the traditional preservation methods studied on the content of micronutrients, antinutritional factors, and level of bioavailability of iron and zinc. The means were segregated using Tukey's honestly significant difference test at 5% level of significance. To determine the contribution of vegetables to household micronutrient intake, the recommended dietary allowance (RDA) of all members in a given household was computed for a day for the selected nutrients considered in this study using RDA tables from Brown et al. (2011). RDA for households was calculated as an aggregate of individual requirements (Okidi et al., 2018). The daily requirements were then standardized to the period of consumption (Brown et al., 2011). The local measurement of cultivated vegetables consumed per day was converted to a standard measure (grams) and computed for the duration over which they were consumed. Using laboratory values determined under Section 2.4, the quantity of nutrients consumed was derived. The contribution of the nutrients from vegetables during the period of consumption was computed as a percentage of the required RDA for each nutrient. Comparison of the contribution to RDA between fresh and preserved samples was undertaken using independent sample *t* test at 5% level of significance (*p* ≤ .05). In all cases, Statistical Package for Social Scientists (SPSS) version 20.0 was used.

## 3 RESULTS

### 3.1 Effect of traditional preservation methods on micronutrient content of vegetables

Data on micronutrient content of vegetables as affected by the various traditional preservation methods investigated are presented in Table 2. Generally, the effect was largely dependent on the type of micronutrient and the type of preservation method. There was a clear distinction between the effects on vitamin A content and the effects observed for mineral micronutrients. For vitamin A, all preservation methods significantly reduced the content of the micronutrient in all the vegetables (*p* ≤ .05) except for *S. melongena* subjected to the salting and sun drying process (*p* > .05). The reduction in vitamin A content was more significant in samples subjected to the boiling and sun drying method than other methods, and occurred at about 50%, 88%, 65%, and 20% for *V. unguicullata*, *A. esculentus*, *H. cannabinus*, and *S. melongena*, respectively.

**TABLE 2:** Micronutrient content (dry weight basis) of vegetables as affected by various traditional preservation methods

Vegetable	Preservation method	Vitamin A (µg RE/100 g)	Iron (mg/100 g)	Calcium (mg/100 g)	Zinc (mg/100 g)	Magnesium (mg/100 g)	Phosphorus (mg/100 g)
*V. unguicullata*	Fresh*	420 ± 0.00^a^	6.62 ± 0.01^a^	3.73 ± 0.04^a^	5.03 ± 0.01^a^	6.63 ± 0.00^a^	134.48 ± 0.24^a^
	Sun drying	300 ± 0.00^b^	6.24 ± 0.01^a^	3.64 ± 0.01^a^	5.23 ± 0.06^a^	6.48 ± 0.09^a^	138.77 ± 0.08^a^
	Boiling and sun drying	220 ± 0.00^c^	1.99 ± 0.00^c^	1.03 ± 0.01^c^	0.91 ± 0.01^c^	1.10 ± 0.02^c^	90.23 ± 0.01^c^
	Salting and sun drying	270 ± 0.00^d^	4.17 ± 1.94^b^	2.09 ± 0.98^b^	3.21 ± 0.01^b^	4.19 ± 0.01^b^	131.92 ± 0.01^b^
*A. esculentus*	Fresh*	80 ± 0.00^a^	9.21 ± 0.00^a^	5.05 ± 0.01^a^	7.43 ± 0.01^a^	4.32 ± 0.01^a^	57.10 ± 0.01^a^
	Sun drying	60 ± 0.00^b^	9.34 ± 0.01^a^	5.30 ± 0.01^a^	7.27 ± 0.06^a^	4.22 ± 0.01^a^	56.80 ± 0.01^a^
	Boiling and sun drying	10 ± 0.00^c^	3.97 ± 0.00^b^	2.76 ± 0.31^c^	2.82 ± 0.01^c^	2.90 ± 0.01^c^	27.24 ± 0.02^c^
	Salting and sun drying	50 ± 0.00^d^	8.37 ± 0.00^c^	3.77 ± 1.03^b^	5.01 ± 0.01^b^	3.15 ± 0.01^b^	40.46 ± 0.01^b^
*H. cannabinus*	Fresh*	430 ± 0.27^a^	4.75 ± 0.01^a^	1.74 ± 0.01^a^	6.51 ± 0.01^a^	2.42 ± 0.00^a^	95.31 ± 0.01^a^
	Sun drying	150 ± 0.00^b^	4.94 ± 0.57^a^	1.69 ± 0.01^a^	6.44 ± 0.11^a^	2.30 ± 0.01^a^	94.81 ± 0.12^a^
	Boiling and sun drying	150 ± 0.00^b^	0.99 ± 0.01^a^	0.44 ± 0.01^c^	1.44 ± 0.01^b^	0.92 ± 0.01^c^	35.24 ± 0.00^c^
	Salting and sun drying	180 ± 0.00^b^	4.01 ± 0.01^d^	1.79 ± 0.01^a^	6.31 ± 0.01^a^	2.42 ± 0.01^a^	80.55 ± 0.01^b^
*S. melongena*	Fresh*	50 ± 0.00^a^	7.21 ± 0.00^a^	2.97 ± 0.02^a^	8.49 ± 0.09^a^	7.39 ± 0.50^a^	193.32 ± 0.11^a^
	Sun drying	30 ± 0.00^b^	7.33 ± 0.11^a^	3.01 ± 0.01^a^	8.62 ± 0.01^a^	7.32 ± 1.00^a^	192.60 ± 0.01^a^
	Boiling and sun drying	40 ± 0.00^c^	3.02 ± 0.01^c^	1.11 ± 0.00^c^	1.89 ± 0.01^b^	2.99 ± 0.01^b^	56.92 ± 0.01^c^
	Salting and sun drying	50 ± 0.00^a^	5.21 ± 0.13^b^	2.20 ± 0.01^b^	8.66 ± 0.01^a^	7.00 ± 0.01^a^	121.70 ± 0.01^b^

Note

Data show mean ± standard deviation (*n* = 6). For each vegetable and for each preservation method, values in the same column followed by different superscripts are significantly different (*p* ≤ .05).

* Fresh: fresh vegetable samples used as control.

In the case of mineral micronutrients, with the exception of the sun drying method, generally all the other preservation methods investigated affected micronutrient contents of the vegetables to various degrees. The effect was largely negative and was registered more in samples subjected to the boiling and sun drying process than in samples treated with the salting and sun drying method (*p* ≤ .05). Irrespective of the vegetable type, the boiled and sun dried samples had significantly lower contents of mineral micronutrients than the salted and sun dried samples (*p* ≤ .05). The reduction in mineral micronutrient contents of the vegetable samples subjected to boiling and sun drying process generally ranged from 25% to 50% except in a few situations were reductions were above 50%. These were largely observed for iron (*S. melongena*: 58%; *V. unguicullata*: 70%; *H. cannabinus*: 79%), zinc (*V. unguicullata*: 82%; *A. esculentus*: 62%; *H. cannabinus*: 78%; and *S. melongena*: 78%), and phosphorous (*S. melongena*: 70%).

### 3.2 Effect of traditional preservation methods on antinutritional factors and nutrient bioavailability

Results showing the effects of various traditional preservation methods on the levels of antinutritional factors in vegetables are presented in Table 3. In general, all the preservation methods significantly reduced the contents of the antinutritional factors investigated (*p* < .05) albeit to various degrees. The level of reduction was dependent on the type of vegetable and the specific antinutritional factor. In terms of vegetable type, the trend and extend of the effect were various. For *V. unguicullata*, the reduction in the level of antinutrients was least for sun drying (total polyphenols: 1%; total oxalates: 60%; tannins: 33%; and phytate: 40%) followed by salting and sun drying (total polyphenols: 47%; total oxalates: 75%; tannins: 67%; and phytate: 63%), and boiling and sun drying (total polyphenols: 52%; total oxalates: 83%; tannins: 77%; and phytate: 75%) in increasing order of magnitude. In the case of *A. esculentus*, a similar trend was observed as was the case for *V. unguicullata* except for the contents of total polyphenols and phytate. The extent of total polyphenol reduction was the same in boiled and sun dried and salted and sun dried samples by about 40% (*p* > .05) while the content of phytate was most reduced in sun dried followed by salted and sun dried and least in boiled and sun dried samples by about 70%, 39%, and 36%, respectively (*p* < .05).

**TABLE 3:** Contents of antinutritional factors in vegetables subjected to various preservation methods

Vegetable	Preservation method	Content of antinutritional factors (mg/100 g)			
		Total phenols	Total oxalates	Tannins	Phytate
*V. unguicullata*	Fresh*	82.55 ± 0.01^a^	50.68 ± 0.15^a^	30.5 ± 0.01^a^	62.47 ± 0.01^a^
	Sun drying	81.60 ± 0.01^b^	20.40 ± 0.23^b^	20.30 ± 0.02^b^	37.33 ± 0.00^b^
	Boiling and sun drying	39.45 ± 0.01^c^	08.55 ± 0.02^c^	07.00 ± 0.01^c^	15.47 ± 0.01^c^
	Salting and sun drying	43.45 ± 0.01^d^	12.67 ± 0.01^d^	10.1 ± 0.01^d^	22.98 ± 0.01^d^
*A. esculentus*	Fresh*	161.40 ± 0.11^a^	54.54 ± 0.32^a^	31.30 ± 0.44^a^	24.43 ± 0.02^a^
	Sun drying	127.55 ± 0.06^b^	25.46 ± 0.73^b^	24.35 ± 0.27^b^	07.25 ± 0.17^b^
	Boiling and sun drying	98.67 ± 0.01^c^	11.63 ± 0.11^c^	12.90 ± 0.02^c^	15.73 ± 0.01^c^
	Salting and sun drying	98.97 ± 0.03^c^	13.01 ± 0.01^d^	13.87 ± 0.02^d^	14.81 ± 0.12^d^
*H. cannabinus*	Fresh*	231.43 ± 0.22^a^	52.74 ± 0.40^a^	34.76 ± 0.03^a^	31.62 ± 0.04^a^
	Sun drying	154.45 ± 0.17^b^	27.30 ± 0.14^b^	24.69 ± 0.01^b^	06.44 ± 0.11^b^
	Boiling and sun drying	88.80 ± 0.01^c^	13.40 ± 0.01^c^	9.99 ± 0.01^c^	12.34 ± 0.01^c^
	Salting and sun drying	95.42 ± 0.01^d^	16.90 ± 0.01^c^	11.39 ± 0.03^d^	14.55 ± 0.01^d^
*S. melongena*	Fresh*	161.47 ± 0.32^a^	49.62 ± 0.15^a^	33.60 ± 0.40^a^	88.78 ± 0.56^a^
	Sun drying	124.38 ± 0.25^b^	20.20 ± 0.16^b^	23.58 ± 0.18^b^	61.11 ± 0.13^b^
	Boiling and sun drying	79.76 ± 0.01^c^	13.92 ± 0.01^c^	13.38 ± 0.02^c^	32.84 ± 0.01^c^
	Salting and sun drying	81.23 ± 0.01^d^	12.88 ± 0.01^d^	15.09 ± 0.01^d^	37.95 ± 0.01^d^

Note

Data show mean ± standard deviation (*n* = 6). For each vegetable and for each preservation method, values in the same column followed by different superscripts are significantly different (*p* ≤ .05).

* Fresh: fresh vegetable samples used as control.

In the case of *H. cannabinus* and *S. melongena*, two scenarios were apparent. First, was the general trend, in qualitative terms for which the extend of reduction in the contents of the antinutrients was highest in boiled and sun dried followed by salted and sun dried and sun dried samples in decreasing order of magnitude (*p* < .05). In quantitative terms, the level of antinutrient reduction was in the range of 53%–75%, 55%–68%, and 23%–48% for boiled and sun dried, salted and sun dried, and sun dried samples, respectively. Secondly, reduction in the content of phytate in *H. cannabinus* and oxalate in *S. melongena* followed different trends. Specifically, the reduction in phytate concentration in *H. cannabinus* was highest for sun dried (80%) followed by boiled and sun dried (61%) and least for salted and sun dried samples (54%) while the retention of oxalate in *S. melongena* was least in salted and sun dried (26%) followed by boiled and sun dried (28%) and highest in sun dried samples (41%) instead.

Table 4 provides data on in vitro bioavailability of iron and zinc from various vegetables subjected to different preservation methods. All the preservation methods investigated improved bioavailability of iron and zinc to various degrees (*p* ≤ .05). The degree of bioavailability improvement was largely dependent on the nutrient and vegetable type. For iron, bioavailability improvement was greatest for samples subjected to boiling and sun drying (*V. unguicullata*: 51%; *A. esculentus*: 52%; *H. cannabinus*: 111%; *S. melongena*: 55%) followed by salting and sun drying (*V. unguicullata*: 39%; *A. esculentus*: 49%; *H*. *cannabinus*: 60%; *S. melongena*: 27%) and then sun drying (*V. unguicullata*: 28%; *A. esculentus*: 38%; *H. cannabinus*: 42%; *S. melongena*: 21%) in decreasing order of magnitude. In terms of preservation method, the greatest and least improvement in iron bioavailability was achieved with *H*. *cannabinus* and *S. melongena* subjected to boiling and drying and sun drying treatment, respectively. With the exception of *H. cannabinus* subjected to the boiling and sun drying process, improvement in iron bioavailability was generally below 65% irrespective of the type of vegetable and the preservation method applied.

**TABLE 4:** In vitro bioavailability of iron and zinc in vegetables preserved using various traditional methods

Nutrient	Vegetable	In vitro bioavailability (%)			
		Fresh*	Sun drying	Boiling and sun drying	Salting and sun drying
Iron	*V. unguicullata*	10.35 ± 0.01^a^	13.23 ± 0.01^b^	15.60 ± 0.01^c^	14.39 ± 0.01^d^
	*A. esculentus*	12.53 ± 001^a^	17.33 ± 0.01^b^	19.00 ± 0.05^c^	18.65 ± 0.02^d^
	*H. cannabinus*	05.18 ± 0.01^a^	07.35 ± 0.02^b^	10.91 ± 0.01^c^	08.31 ± 0.01^d^
	*S. melongena*	06.23 ± 0.01^a^	07.52 ± 0.02^b^	09.67 ± 0.00^c^	07.90 ± 0.01^d^
Zinc	*V. unguicullata*	04.05 ± 0.01^a^	05.75 ± 0.01^b^	07.55 ± 0.001^c^	07.01 ± 0.01^c^
	*A. esculentus*	03.71 ± 0.01^a^	09.28 ± 0.01^b^	10.99 ± 0.01^c^	09.89 ± 0.51^b^
	*H. cannabinus*	02.18 ± 0.01^a^	05.55 ± 0.01^b^	08.48 ± 1.99^d^	07.90 ± 0.01^c^
	*S. melongena*	03.06 ± 0.01^a^	06.00 ± 0.01^b^	08.45 ± 0.02^c^	07.00 ± 0.01^d^

Note

Data show mean ± standard deviation (*n* = 6). For each vegetable and for each nutrient, values in the same row followed by different superscripts are significantly different (*p* ≤ .05).

* Fresh: fresh vegetable samples used as control.

In the case of zinc, improvement in bioavailability also followed the same trend as was observed in the case of iron. Improvement in zinc bioavailability was lowest in sun dried (*V. unguicullata*: 42%; *A. esculentus*: 165%; *H. cannabinus*: 155%; *S. melongena*: 96%) followed by salted sand sun dried (*V*. *unguicullata*: 73%; *A. esculentus*: 167%; *H*. *cannabinus*: 262%; *S. melongena*: 129%) and boiled sand sun dried (*V. unguicullata*: 86%; *A. esculentus*: 196%; *H. cannabinus*: 289%; *S. melongena*: 176%) in increasing order of magnitude, except equality between *A. esculentus* samples subjected to salting and sun drying (167%) and sun drying (165%). Unlike for the scenario observed regarding iron bioavailability, in the case of zinc, bioavailability improvements were generally above 100% except for *V. unguicullata* and *S. melongena* samples subjected to sun drying process, and *V. unguicullata* samples treated through a process of salting and sun drying.

### 3.3 Effect of traditional preservation on the contribution of vegetables to household micronutrient requirements

**FIGURE 2:**
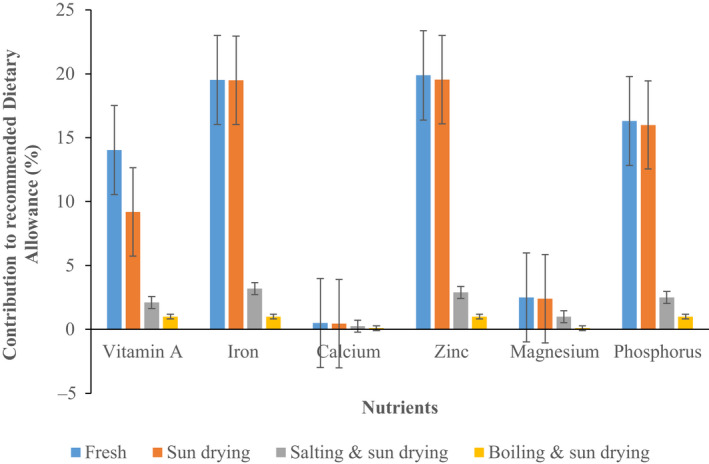
Contribution (%) of vegetables preserved using various traditional preservation methods to household micronutrient requirements (Recommended Dietary Allowance, RDA). Calculation of contribution based on RDA for the period of consumption

Data showing the effect of various traditional preservation methods on the contribution of vegetables (all vegetables combined) to household requirement (RDA) for vitamin A, iron, calcium, zinc, magnesium, and phosphorus are presented in Figure 2.

The effect was dependent on type of nutrient and preservation method applied. Sun drying significantly reduced the contribution of vegetables to household RDA for vitamin A by about 35% (*p* < .05) but had no effect on the contribution toward the intake of mineral micronutrients (*p* > .05). All the other preservation methods significantly reduced the contribution of vegetables to household RDA for all the micronutrients to various degrees except for calcium. Generally, in situations where reductions were significant, the extent was more for boiled and sun dried vegetables than those preserved using salting and sun drying. Boiling and sun drying reduced the contribution to RDA for vitamin A, iron, zinc, magnesium, and phosphorus by 93%, 83%, 75%, 58%, and 94%, while the salting and sun drying reduced the contribution by 85%, 77%, 85%, 60%, and 85%, respectively.

**FIGURE 3:**
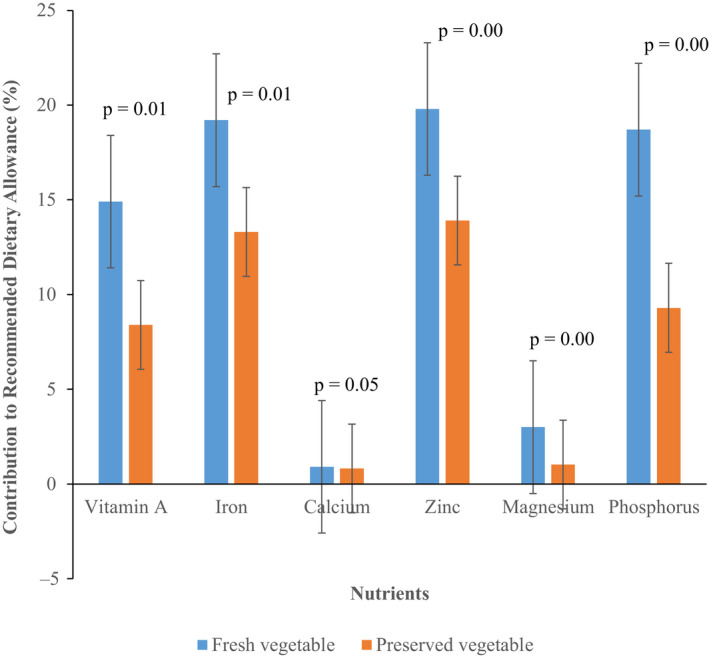
Combined effect of traditional preservation methods (sun drying, salting and sun drying, boiling, and sun drying) on the contribution (%) of selected cultivated vegetables to household micronutrient requirements (Recommended Dietary Allowance: RDA). Calculation of contribution based on RDA for the period of consumption

The combined effect of the three traditional preservation methods on the contribution of the vegetables to household RDA for vitamin A, iron, zinc, phosphorus, magnesium, and calcium is presented in Figure 3. The three traditional methods reduced the contribution of vegetables to household RDA for vitamin A, iron, magnesium, and phosphorous (*p* < .05) but marginally for calcium (*p* = .05). The reduction was highest for magnesium (61%), followed by vitamin A (60%), phosphorus (54%), iron (32%), and zinc (28%) in decreasing order of magnitude.

## 4 DISCUSSIONS

### 4.1 Effect of traditional preservation methods on micronutrient content of vegetables

Fundamentally, food preservation is an essential element that affects the utilization dimension of food and nutrition security (Amit et al., 2017; Bourdoux et al., 2016). It is pertinent for global food supply but critical particularly in localities where food production is dependent on natural weather conditions and seasonality (Ayua & Omware, 2013). Nonetheless, it is important to appreciate that for food preservation to effectively contribute to food and nutrition security, the preservation methods applied should not compromise substantially the nutritional content of the foods. Literature is replete with information on standardized preservation methods such as freezing, canning, pasteurization, refrigeration, high hydrostatic pressure, and microwave cooking (Barrett & Lloyd, 2012). In rural set‐ups typical of many developing countries, traditional preservation methods are dominantly used (Hassan et al., 2007; Santhi & Sengottuvel, 2016) and the manner of their application vary from location to location dictated by culture and tradition (Alowo et al., 2018; Asogwa et al., 2017). Due to their significance in off‐setting seasonality constraints in food production, the efficacy of traditional preservation technologies on quality and availability of nutrients needs to be understood. However, this requires empirical data on various traditional methods as applied in practice.

The results have demonstrated that the three preservation methods investigated affected the micronutrient content of the vegetables to various extends. Boiling and sun drying had the most negative impact irrespective of the nutrient type. Mineral micronutrients are largely water soluble (Sheahan & Barrett, 2017), and therefore, the significant reduction in mineral contents of the boiled and sun dried samples could be largely due to leaching (into the boiling water) although mineral contents in the leachate was not measured. Sun drying did not significantly affect the mineral micronutrients. This is because sun drying largely reduces the moisture content of vegetables but does not affect the mineral contents which are stable to heat (Hassan et al., 2007). This is also well‐illustrated with the results from this study which shows that sun drying alone had no effect on mineral contents of the vegetables. Losses of mineral micronutrients in water used during cooking through leaching have been demonstrated by various laboratory studies (Babalola & Alabi, 2015; Lewu et al., 2009), and because of this fact, it is normally recommended that water used for cooking vegetables should constitute part of the meal and should not be discarded (Oluoch et al., 2012). In the context of vegetable preservation through a process of boiling and sun drying, the final stable product comes out in a dry form. A key question that still needs to be answered is how to recover, under rural conditions, the minerals lost in the leachate to be added back to the final dry product. This is a potential subject for future research.

Significant loss of vitamin A from vegetables was registered following the application of boiling and sun drying process. Vegetables contain largely β‐carotene (the precursor of vitamin A) which is largely insoluble in aqueous system (Musa & Ogbadoyi, 2012). Therefore, losses recorded for vitamin A cannot be attributed to leaching as adduced for the case of mineral micronutrients, but rather due to heat inactivation during boiling and photo‐oxidation during the drying process. β‐carotene is heat labile (Oluoch et al., 2012) and sensitive to photo‐oxidation. Therefore, the combination of heat treatment and sunlight could have caused oxidation of the carotenoids in the vegetables to a compound with no beta‐carotene activity (Musa & Ogbadoyi, 2012). On the other hand, moist heat treatment of vegetables (blanching) is a pretreatment action essential for inactivating endogenous enzymes (such as lipoxygenase) that cause off‐flavors during storage (Workneh et al., 2014). Industrially, blanching is well optimized for nutrient retention for conventional vegetables such as carrots, peas, and beans (Miglio et al., 2008). However, in the context of this study, whereas boiling prior to sun drying could be essential for inactivating enzymes, it is apparent that the process as applied traditionally leads to excessive loss of essential micronutrients (25%–82%). Future studies should optimize the traditional heat treatment process to minimize nutrient losses from vegetables. Interestingly, it seems reduction in micronutrient content of vegetables subjected to boiling and sun drying is not a general phenomenon. This is because, whereas results of other studies conducted under laboratory conditions using *chaya* leaves, *Cnidoscolus aconitifolius* (Adanlawo & Elekofehinti, 2012; Babalola & Alabi, 2015; Oduse et al., 2012), concur with the findings from the current study, the work of Lewu et al. (2009) indicated noneffect based on experiment conducted using *Colocasia esculenta*.

Salting and sun drying method also caused significant losses of vitamin A and mineral contents of the vegetables but the effect was second to boiling and sun drying method in terms of magnitude. With respect to mineral micronutrients, sun drying alone had no effect on their contents in vegetables. This implies that the low levels of mineral micronutrients observed in salted and sun dried vegetables were caused by the action of salt. Sodium a major ingredient in table salt causes tissue weakening and destruction (Miglio et al., 2008). Thus, reduction in the levels of minerals could probably be attributed to drip loss caused by tissue weakening and injury. However, empirical evidence is necessary to confirm this assertion by assessing the extent of the drip and the amount of minerals, if any in the drip pool. This is another potential subject of future research. Loss of β‐carotene in vegetables due to photo‐oxidation is well known (Kolawole et al., 2011). Results from this study shows that loss of vitamin A was more from vegetables subjected to salting and sun drying than those exposed to sun drying process. This suggests that salt had an effect. However, the mechanism of β‐carotene loss due to action of salt is largely unknown. This is another potential subject for future research.

Among all the preservation methods investigated, sun drying was the most effective in ensuring retention of mineral micronutrients in the vegetables. This result contrasts sharply with findings from other studies. For instance, Zoro et al. (2015) reported higher content of mineral micronutrients in vegetables while results of Musa and Ogbadoyi (2012) and Oni et al. (2015) illustrated an inverse situation. It is important to appreciate that the results of these studies cannot directly be compared with the findings from the current study due to methodological disparity. In the current study, all measurements were conducted and reported on a dry weight basis while in other studies the bases for nutrient content measurements were not explicitly indicated. It was clear that the extent to which various preservation methods reduced the contents of micronutrients varied with the type of vegetable. This indicates that the rate of loss of micronutrients from the vegetables varies from species to species. The observed variations could be due to differences in botanical architecture of various plant species. Future studies aimed at optimizing the traditional preservation methods for optimal nutrient retention should take due consideration of interspecies differences.

### 4.2 Effect of traditional preservation methods on antinutritional factors and nutrient bioavailability

The occurrence of antinutritional factors in vegetables limits bioavailability of mineral micronutrients from them (Mepba et al., 2007). Thus, innovations in food processing to improve bioavailability of essential minerals such as iron and zinc have been explored (Ertop & Bektaş, 2018; Mbah et al., 2012). In those explorations, limited attention has been paid to traditional processing methods such as those investigated in this study. Interestingly, the results of this study have demonstrated that the three traditional preservation methods significantly reduced the contents of the four antinutritional factors in all the four vegetable species used. This illustrates the potential of the traditional food preservation technologies to address not only the seasonality and perishability constraints, but also to improve mineral nutrition among rural households. These results are in line with but also in disagreement with the findings from other studies depending on the preservation method. With regard to sun drying method, some studies have reported reduction in the level of tannins, total oxalates, and phytates in vegetables (Abiodun & Adepeju, 2011; Matazu & Haroun, 2004; Musa & Ogbadoyi, 2012; Oni et al., 2015) and are in agreement with the results of the current study. However, the levels of reductions reported in the quoted studies differ with the levels recorded from the current study. These differences could be due to differences in how the traditional technologies are applied in different localities dictated by tradition as well as taste and preferences accorded to the end‐products (Barrett & Lloyd, 2012; Oni et al., 2015). The differences could also be due to differences among vegetable species or cultivars used occasioned by differences in botanical micro‐structural arrangements. For instance, Oni et al. (2015) used *T. occidentalis*, *B. alba*, *A. hybridus*, *T. triangulare*, *C. biafrae*, and *C. rubens* while the current study used *V. unguicullata*, *A. esculentus*, *H. cannabinus*, and *S*. *melongena*. Contrasting results of the current study with respect to sun drying is the work of Zoro et al. (2015) which shows an increase in total phenols after sun drying five common Ivorian vegetables (*A. esculentus*, *Celosia argentea*, *Ipomea batatas*, *Manihot esculenta*, and *Myrianthus arboreus*). There is no apparent explanation for the observed inverse between the results of the current study and that of Zoro et al. (2015) because an increase in phenolic content would suggest de novo synthesis of polyphenols during the drying process, a process which was not measured in either studies. In addition, for both studies, all measurements were conducted and recorded on dry weight basis. On the other hand, if measurements were made on wet weight basis, the reported increase would be due to concentration effect as the water evaporates from the vegetables (Lewu et al., 2009).

Boiling and sun drying, and salting and sun drying reduced all the antinutrients but the decline was more in the boiled and sun dried, followed by the salted and sun dried and least for the sun dried forms. This trend suggests that moist heat and salt had substantial negative effects on the antinutritional factors investigated because the combined effect of boiling and sun drying or salting and sun drying was much higher than sun drying alone. For the boiling and sun drying process, during moist heat treatment of the vegetables, their cell walls might have been destroyed which caused leaching of the contents (Adeleke et al., 2017). The antinutritional factors investigated are water soluble (Oni et al., 2015) and could have been lost through leaching into the boiling water. Similarly, other studies (Babalola & Alabi, 2015; Luo & Xie, 2013) reported comparable results when *Vicia faba* L. and *C. aconitifolius* were subjected to the boiling and sun drying process. In addition, as adduced by Blaabjerg et al. (2010) and ElMaki et al. (2007), moist heat exposure at a temperature of 40–55°C degrades inositol hexaphosphate to the pentaphosphate or lower molecular weight forms thus lowering its concentration. More so, the phytic acid content decreased during boiling probably because insoluble complexes between phytate and other compounds were formed thereby reducing the amount of free phytates (Mazahib et al., 2013).

Superimposing the results of nutrient retention on the results for antinutrients provides an interesting scenario. On nutrient retention, sun drying was the best, followed by salting and sun drying, and least with boiling and sun drying. However, for antinutritional factors, the reverse was true. Generally, in food preservation, the aim among other issues is to inactivate antinutritional factors but observing minimal nutrient losses (Adeleke et al., 2017; Barrett & Lloyd, 2012). This implies that traditional preservation technologies should be optimized. Future studies should consider this aspect. Antinutritional factors investigated in this study such as phenolic acids have free radical scavenging potentials and as such could therefore contribute to lowering cellular aging process in human body (Xu et al., 2017) and enhance immune function (Pereira et al., 2016). Because the preservation methods investigated substantially reduced the content of antinutritional factors examined, it is plausible that traditional preservation methods impacted on the antioxidative potential of the vegetables used in the study, but empirical evidence is required. This is another potential subject for future studies.

Iron and zinc are among the trace minerals of greatest concern when considering the nutritional value of vegetarian diets (Ertop & Bektaş, 2018; Sobiecki et al., 2016) and nutritional needs of women of reproductive age and children in developing countries (Sharma & Dhandoria, 2019; Wessells & Brown, 2012). This situation provides a strong rationale for improving bioavailability of iron and zinc from plant‐based foods. There exists an inverse relationship between bioavailability of essential nutrients such as iron and zinc and the contents of antinutritional factors in plant‐based foods (Adetuyi et al., 2011; Afify et al., 2011). Therefore, processing/preservation technologies that reduce the content of antinutritional factors are expected to improve bioavailability of iron and zinc (Barrett & Lloyd, 2012). Results of this study have demonstrated that in vitro bioavailability of iron and zinc was highest in the boiled and sun dried than in salted and sun dried, and least in sun dried samples compared to fresh samples. This is in tandem with results on antinutritional factors which show that their contents were most reduced by boiling and sun drying, followed by salting and sun drying and least by sun drying. Looking at the results from the element of nutrient retention through inactivation of antinutrients to bioavailability, it is apparent that boiling and sun drying is the best at improving bioavailability of iron and zinc but also causes most significant loss of micronutrients. Therefore, improvements in bioavailability of iron and zinc can be attributed to reduction in the levels of antinutritional factors investigated in this study. A key question that remains unanswered is how the improved nutrient bioavailability of micronutrients can be balanced against losses. The same scenario applies to the salting and sun drying method. On the other hand, improvement in in vitro bioavailability of iron and zinc was not uniform across all the vegetables investigated, but varied among them. This suggests variability factor based on the botanical differences among the plant species used in the study. Improvements in bioavailability observed in this study are comparable to that of Samia et al. (2005) and in some situations much better than those reported by Ertop and Bektaş (2018) and Gupta et al. (2006) in studies conducted under controlled laboratory conditions. This again indicates the potential of traditional preservation technologies at improving bioavailability of mineral micronutrients from plant‐based foods and further justifies the need for the current study. Above all, lack of harmony in the level of results reported in the current study and others reported before implies that more work still needs to be done in order to fully understand how traditional food preservation technologies impact on nutrient content, content of antinutritional factors, and bioavailability of nutrients from vegetables under rural conditions. In addition, future studies should address the issue of bioaccessibility, an important aspect of nutrition which was not considered in this study.

### 4.3 Contribution of cultivated vegetables to household micronutrient nutrition

In rural areas in developing countries, vegetables are the main source of micronutrients because animal source foods, despite having high‐quality nutrients in terms of bioavailability, are too expensive to be included regularly in the diet (Boukary et al., 2016; Oryema et al., 2015). Despite their importance, the significance of vegetables to household nutrition in rural areas is largely recognized in qualitative terms. However, the contribution of vegetables to household nutrient needs in terms of the RDA for micronutrients has not been adequately quantified. Okidi et al. (2018) showed that wild foods contributed more than adequate amount of vitamin A and iron to household nutrition in Acholi subregion of Uganda, however, for cultivated vegetables information lacuna exists. This information is necessary for fostering regular vegetable consumption to enable adequate intake of essential micronutrients.

Generally, the results of the current study show that the four main cultivated vegetables used in the study contributes between 14% and 20% of the household RDA for essential micronutrients when consumed in the fresh form. However, when processed, the contribution reduces to various degrees for various micronutrients except for mineral micronutrients from vegetables processed using sun drying method. However, as observed for other aspects investigated in this study (nutrient retention, content of antinutritional factors, and bioavailability of iron and zinc), boiling and sun drying have the most negative effect on the contribution followed by salting and sun drying. In totality, whereas preservation methods investigated improve bioavailability of nutrients from the vegetables, they potentially reduce the contribution of vegetables to household RDA for essential micronutrients ostensibly due to nutrient losses. The observed substantial reduction in the contribution of the preserved vegetables to the RDA may not only be due to nutrient losses due to preservation but consumption of preserved vegetables sparingly over a longer duration of time. It also suggests that those vegetables that had higher nutrient losses dominated vegetable intake during the lean season. This implies that whereas, food preservation in principle, is essential for reducing food wastage and overcoming scarcity during off‐season (Kiremire et al., 2010), traditional preservation technologies as applied by rural households compromises nutrition security due to the observed high level of nutrient losses especially for boiling and sun drying, and salting and sun drying.

As already noted under Sections 3.1 and 3.2 of this paper, it is important to reiterate the opinion that traditional preservation methods require optimization to minimize nutrient losses to enable rural people take advantages associated with improved nutrient bioavailability from preserved vegetables. A critical search through literature reveals that this is the first time the effect of traditional preservation technologies on the contribution of cultivated vegetables to household RDA for the micronutrients has been studied. However, from a methodological point of view, it is apparent that not all vegetables that can potentially be preserved were included in the study. Therefore, the 14%–20% contribution reported in the current study may be an underestimation. This is because food choice is largely determined by taste and other sensory appeals but rarely informed by nutritional content nor nutritional quality (Flyman & Afolayan, 2006; Mavengahama et al., 2013). It is plausible that other vegetables not included in the study could contain nutrients in superior quantities although not regularly preferred by rural households. Future studies should extend the scope of vegetables types.

## 5 CONCLUSIONS

The results of this study have demonstrated that traditional African vegetable preservation technologies have mixed effects (desired and undesired). Whereas the ultimate desired outcomes of any food preservation strategies are to extend shelf life, improve nutritional quality and guarantee safety, in the current study, improvements in bioavailability of essential nutrients (iron and zinc) were highly associated with significant loss of nutrients instead, culminating in significant reduction in the contribution of cultivated vegetables to household RDA for micronutrients. Therefore, traditional African vegetable preservation methods should be optimized for nutrient retention to enable households derive more micronutrients from vegetables.

## CONFLICT OF INTEREST

The authors declare no conflict of interest.

## ETHICAL REVIEW

Ethical clearance was obtained from Gulu University Research Ethics Committee (GUREC 008–18).

## INFORMED CONSENT

Informed consent was sought at household level from the eligible study participants who were assured of confidentiality of the information provided, and their names were not recorded on the questionnaire. Participants were free to ask questions and terminate the interview if they so wished.

## Data Availability

The data that support the findings of this study have already been included in the manuscript. Raw data are available from the corresponding author upon reasonable request.
